# Prehospital Predictors for Urgent Neurosurgical Intervention in the Head Trauma Patient: A 2-Year Multicenter Retrospective Study

**DOI:** 10.1155/2023/5571435

**Published:** 2023-08-18

**Authors:** Jakub Nożewski, Maja Kwiatkowska, Aleksandra Łosińska, Ewa Nowak, Anna Lackowska, Anna Rydzewska, Agnieszka Durma, Paweł Sokal

**Affiliations:** ^1^Department of Emergency Medicine, Dr John Biziel's Clinical University Hospital No. 2, Bydgoszcz, Poland; ^2^Medical University of Gdańsk, Gdańsk, Poland; ^3^Department of Radiology and Diagnostic Imaging, Dr Antoni Jurasz Clinical University Hospital No. 1, Trauma Center, Bydgoszcz, Poland; ^4^Department of Neurosurgery and Neurology, Dr John Biziel's Clinical University Hospital No. 2, Bydgoszcz, Poland; ^5^Faculty of Health Science, Collegium Medicum in Bydgoszcz, Nicolaus Copernicus University in Toruń, Bydgoszcz, Poland

## Abstract

**Background:**

Traumatic brain injury (TBI) is the main cause of disability in the world. Prehospital diagnosis of patients requiring rapid neurosurgical intervention and the earliest possible introduction of procedures preventing secondary brain injuries (SBI) are crucial. *Methodology and Study Population*. The authors of this paper assumed that certain age groups with specific injuries are more likely to require urgent neurosurgical intervention compared with patients who did not require such an intervention. Out of 54,814 head CT scans, based on the inclusion criteria, 7,864 were selected for the study. Data such as sex, age, the mechanism of injury, comorbid trauma, and abnormal findings in the examination of patients qualified for urgent neurosurgical intervention were analyzed in order to find statistically significant factors through a comparison with all head trauma patients.

**Results:**

Patients qualified for urgent neurosurgical intervention were significantly older compared with the others (63 years vs. 49 years). Patients transferred from the emergency department directly to the operating room were more often admitted to the hospital due to the fall (64.1% vs. 45.1%, *p* = 0.004). The following were observed much more commonly among the patients qualified for urgent neurosurgical intervention than in the entire study group of subjects with traumatic brain injury (TBI), e.g., calf deformity (2.2% vs. 0.1%, *p* = 0.019) and bleeding from the mouth (4.3% vs. 0.0%, *p* < 0.001). On the other hand, superciliary arch wounds were observed much less commonly than in the entire group (0.0% vs. 5%, *p* = 0.221).

**Conclusion:**

Patients admitted directly to the operating neurosurgical room from emergency departments constitute a small percentage of TBI patients, and their prognosis for normal performance status upon discharge is poor. Maximum efforts should be made to distinguish these patients and to start proper treatment even during prehospital care.

## 1. Introduction

Head trauma is an important and growing public health problem. Sometimes it is even referred to as a silent pandemic [1]. Traumatic brain injuries (TBIs) are the main cause of disability among healthy men around the world. In the USA alone, there were over 64,000 TBI-related deaths in 2020, which equates to 176 TBI-related deaths every day [2]. It is estimated that every year, 500–800 out of every 100,000 European residents will suffer from head trauma. As these data come primarily from developed countries, researchers suppose that these numbers may be three times higher in China and other developing countries [3]. Aging population is another problem. Until recently, TBI patients were mostly young men involved in road traffic collision (RTC), while the current data show that almost one-third of TBI patients are people aged over 75, who suffer a fall. This age group also accounts for 32% of TBI-related hospitalizations and 28% of deaths [4]. Therefore, each year an estimated 69 million individuals will suffer a TBI, the vast majority of which will be mild (81%) or moderate (11%) in severity. Per capita, the highest annual incidence of all-cause TBIs is observed in the North America and Europe (1,299 and 1,012 cases per 100,000 people, respectively) [5]. It seems that knowledge about how to stop a pathophysiological trauma-related reaction in the brain and especially an effective and evidence-based medicine (EBM) treatment is disproportionately low. According to data from the current Brain Trauma Foundation guidelines, none of the work has produced even one valid conclusion, pharmacotherapy, or procedure improving TBI management in the last 20 years. Since most work does not provide any evidence, interest in research on this issue is declining among sponsors and pharmaceutical companies. For this reason, ongoing assessment of TBI patients admitted for neurosurgical treatment seems to be crucial. The authors decided to examine the demographic and clinical characteristics and the mechanism of trauma, which could indicate the need for urgent neurosurgical intervention at the stage of prehospital medicine.

### 1.1. Study Population

**Figure 1:**
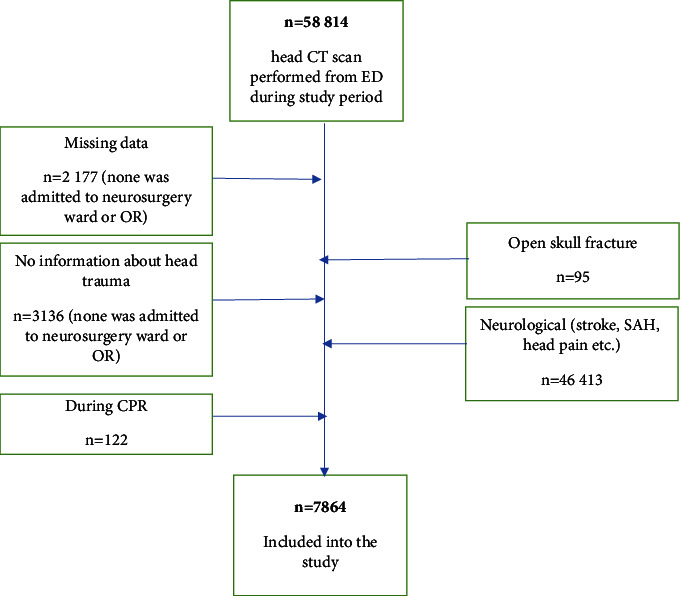
Patients admitted to Emergency Medicine Departments of two clinical hospitals who underwent head CT scan.

We retrospectively evaluated data from two clinical university hospitals from head trauma patients in 2018-2019. As the authors were aware of the risk that many CT results could be omitted if an appropriate ICD-10 S code was missing, the decision was made to request a list of all head CT scans ordered at the ED level from the authorities of both hospitals. In total, 54,814 results were received (29,141 CT scans from a trauma centre and 25,673 head CT scans from another university hospital, which is also the only thrombectomy center within a 100-km radius). All 54,814 hospital records of patients admitted to both EDs were analyzed. In any case where basic data on the reason for CT were missing, data were excluded (Figure 1). A total of 7,930 ED visits were obtained after applying the inclusion criteria: age >18 years, closed head trauma, patient delivered by an emergency medical team, referred, or presenting on their own due to head trauma, mention of head trauma in the emergency medical records or in the referral, signs of head trauma on physical examination, and preserved vital functions upon admission. The exclusion criteria were no signs of trauma on examination, no mention of head trauma in the medical record, stroke, and patients who underwent cardiopulmonary resuscitation before reaching hospital.

### 1.2. Statistical Analysis

The Mann–Whitney *U* test, Kruskal–Wallis test (with Dunn's post hoc test and correction for multiple Bonferonni testing), chi-square, and Fisher tests were used in the study. The Mann–Whitney *U* test is a nonparametric test used to compare numerical variables between two groups of observations. Statistically significant results obtained on its basis prove that there is a difference in the distribution of a given variable between these groups. The Kruskal–Wallis test is also a nonparametric test for comparing the distribution of a variable between multiple groups. In order to examine the relationship between categorical variables, the chi-square test or Fisher's test was used. The significance level was *p*=0.05; however, statistically significant results were also indicated for the levels of *p*=0.01 and *p*=0.001.

## 2. Results

Table 1 shows general patient characteristics. Males comprised slightly more than 60% of the patients (*n* = 4753, 60.3%), while females nearly 40%. The median age was 49.3 years; the standard deviation was over 42% of the mean value, which indicates significant age diversity. On an average, women were 55 years of age, while the mean age of men were 45.5 years. The minimum age was identical in both groups (18 years), while the maximum age was slightly higher in women (104 years). Based on the analysis, it can be stated that there was a statistically significant age difference between men and women (*p* < 0.05). The analysis of the weekly distribution of patients revealed that in general, most patients were admitted on Mondays (15.0%), Sundays (14.9%), and Thursdays (14.9%). The fewest were admitted on Wednesdays (13.0%). No statistically significant differences were observed between individual years and months. After implementation of inclusion criteria, all 7,864 patients were divided into 5 groups: (1) those discharged directly from ED or admitted to nonneurosurgical ward, (2) C1—qualified for exigent neurosurgical intervention–direct transfer from ED to operating room, (3) C2—qualified for neurosurgical intervention <24 hours, (4) C3—admitted to the Department of Neurosurgery or observed in ED for 24 hours in order to perform a control head CT scan and assess the progression/regression of posttraumatic lesions, (5) C4—no neurosurgical indication for hospitalization but patients were on anticoagulants, dangerous mechanism of trauma, were severely vomiting, confused, or/and without good family care.

For each patient, the main mechanism of trauma was determined and assigned to one of the 13 categories. Patients were most commonly hospitalized because of a fall (46.4%), RTC (16.9%), and beating (13.2%). Less common causes included hitting (9.4%), fainting (5.7%), epilepsy (5.7%), and pedestrian hit by a car (1.9%). The other categories accounted for less than 1% of trauma mechanisms. All individual cases were classified into the category “other” (rape was an exception distinguished separately). Almost 21% of the patients did not present at the hospital at the day of injury. The majority presented between one and four days following the injury. However, some patients waited much longer, which can be seen by a large difference between the mean (about 4.5 days) and median (2 days) as well as a wide spread of observations (SD of about 12 days). The median Glasgow Coma Scale (GCS) score was 15. Loss of consciousness was reported in slightly over 42% of the patients, and 0.5% recalled the event only partially. Over 21% of the patients were admitted under the influence of alcohol, with a mean level of about 304 (±107) mg/dL. Neurological abnormalities were found in 7.1% of the patients, with anisocoria being the most common (19.7%). Confusion (13%), paresis of the limb (8.1%), and amnesia (7.3%) were less common. Complaints were reported in 48% of the patients. Among them, the average number of complaints was two (±1). Physical examination showed abnormalities in over 65% of the patients. One or two abnormalities were usually found, although the record number was 10 in some cases. Other radiological examinations (apart from head CT that qualified patients for the study) were performed in almost 60% of the patients and included CT angiography (0.2%), X-ray (44%), CT (28.8%), and ultrasound (13.6%). It is worth noting that trauma CT was performed in 1.3% of the patients. Radiological examinations revealed 74 different abnormalities. The most common were nasal fracture (4.6%), orbital fracture (2.9%), maxillary sinus fracture (2.6%), and fracture of at least one rib (2.4%). Detailed analysis is shown in Table 2.

**Figure 2:**
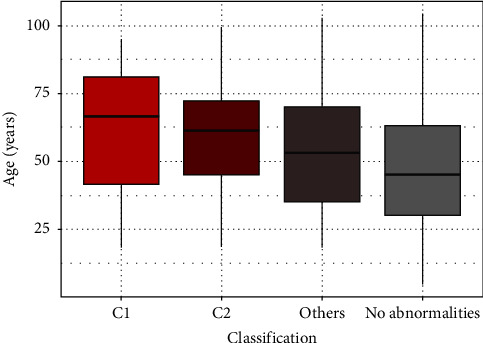
Relationship between the variables: age (years) and classification.

Patients qualified for urgent neurosurgery (C1) were significantly older compared with the others (mean of about 63 years vs. 49 years). However, no significant differences were found between the C1 and C2 groups, meaning that patients with delayed surgery were not significantly younger than those qualified for urgent neurosurgical intervention (63.31 vs. 58.76). There is a visible trend of a higher risk of more severe abnormalities in older patients; mean age changes depending on the group (C1: 63 years, C2: 59 years, others with diagnosed injury: 53 years, and no injury: 48 years). Age differences between the groups are presented in Figure 2. However, it is worth noting that SD values of the age are quite similar in these groups. C1 patients who were admitted to the hospital on a day other than the day of injury presented significantly later than patients in the other groups (mean, 5.31 days vs. 4.67 days; median, 3.5 days vs. 2 days), including those in the C2 group (mean, 5.31 days vs. 2.68 days; median, 3.5 days vs. 2 days). The long period before arrival at the hospital from the time of the injury in patients qualified for urgent neurosurgical intervention resulted from the patient's refusal to transfer to the hospital immediately after the injury, explaining drowsiness/decrease in GCS with possible alcohol intoxication or loneliness and rare contact with the family. Which was easy to predict, GCS in C1 patients group had significantly lower scores than patients in the other groups (mean, 10.68 vs. 14.83; median 13 vs. 15), including those in the C2 group (mean, 10.68 vs. 12.99; median 13 vs. 15). The proportion of patients who lost consciousness was higher in the C1 group than in the other groups (67.7% vs. 42%) with no significant differences between the C1 and C2 groups. Data are summarized in Table 2.

Patients qualified for instant transfer to the operating room were more often admitted to the hospital due to the fall (64.1% vs. 45.1%). Less common causes were RTC (4.7% vs. 16.5%) and beating (4.7% vs. 13.0%). Significance results for individual trauma mechanisms are shown in Table 3. To verify between which mechanisms of trauma the groups differed significantly, tests for differences in proportions were carried out. Such an analysis showed that statistical significance (*p* value <0.05) was achieved for the fall and road traffic accident. The former was more common in the C1 group than in the other groups (64.1% vs. 45.1%), while the latter less common (4.7% vs. 16.5%). To verify differences between all patients who had neurosurgery intervention (<24 from transfer to hospital) with patient without neurosurgery during hospital stay, between which mechanisms of trauma and the groups differed significantly with tests for differences in proportions were carried out again (C1 + C2 group vs. rest of the patients). This analysis showed that statistical significance (*p* value <0.05) was achieved for the fall and RTC. The former was more common in the C1 and C2 groups than in the other groups (61.5% vs. 46%), while the latter less common (10.4% vs. 17.1%). Differences were also observed for beating (6.5% vs. 13.5%) and hitting (3.9% vs. 9.6%), which occurred less frequently in the C1 and C2 groups than in the others. In contrast, epilepsy-related injuries were more common in the C1 and C2 groups (10.8% vs. 5.5%).

**Figure 3:**
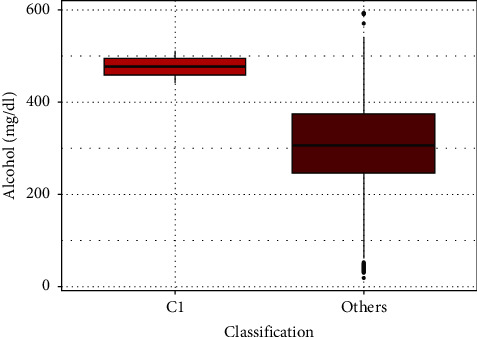
Relationship between the variables: alcohol (mg/dL) and classification.

The number of patients under the influence of alcohol between C1 and all other head trauma patients' groups was comparable and with no statistically significant difference. However, C1 patients under the influence of alcohol had higher mean blood alcohol levels than patients from other groups (mean, 476 mg/dL vs. 303.98 md/dL; median 476 mg/dL vs. 306 mg/dL) (Figure 3). The alcohol level was associated with a significantly lower risk of death. Using the test for differences in proportions, the occurrence of neurological abnormalities was compared between C1 patients and all the other patients. Significant differences (*p* value <0.05) are summarized in Table 4.

Neurological abnormalities were significantly more commonly reported in C1 group patients (71.9% vs. 39.9%). The C1 and C2 groups did not differ significantly in specific neurological abnormalities (*p* value >0.05). Every patient admitted for urgent neurosurgical intervention had a concurrent injury of another body part. Compared with other patients, a significantly higher percentage of patients admitted for urgent neurosurgical intervention (C1) had the following: deformity of the lower leg, bleeding from the mouth, bleeding from the nose, hematoma in the parietal region, wound of the occiput, wound in the temporal region, contusion of the shoulder, contusion of the occiput, and palpable skull fracture. Among the complaints, significant differences were found for pain in the cervical spine/neck which was uncommon in the C1 group (0.0% vs. 12.2%). The following Table 5 shows signs and symptoms classified into those found on physical examination and those reported by the patients. There were a lot of complaints significantly differentiating the C1 and C2 groups from all the other head trauma patients. These included the following:

- More common in C1 + C2: convulsions with loss of consciousness (0.5% vs. 0%, *p*=0.004), bleeding from the ear (4.2% vs. 0.1%, *p* < 0.001), bleeding from the mouth (1.6% vs. 0%, *p* < 0.001), bleeding from the nose (3.7% vs. 1.1%, *p* < 0.004), periorbital ecchymosis (3.7% vs. 1%, *p*=0.002), bilateral periorbital ecchymosis (8.4% vs. 4.9%, *p*=0.044), wound of the occiput (12% vs. 5.4%, <0.001), contused wound in the temporal region (1.6% vs. 0.2%, *p*=0.002); contusion of the hand (2.1% vs. 0.6%, *p*=0.049), and palpable skull fracture (2.6% vs. 0.1%, *p* < 0.001).
- More common in the other group than in C1 + C2: wound of the superciliary arch (1.6% vs. 5.1%, *p*=0.041);

Among the complaints, significant differences were found for the following sings and/or symptoms: Pain in the cervical spine/neck–less common in C1 and C2 patients than in the other patients with any signs and/or symptoms (3.7% vs. 12.3%, *p* < 0.001), vomiting–more common in C1 and C2 patients than in the other patients with any signs and/or symptoms (7.9% vs. 3.7%, *p*=0.006). Death occurred more frequently in C1 patients than the rest of the patients (32.8% vs. 0.6%) and more often in the C1 or C2 groups than the rest of the patients (19.5% vs. 0.2%).

## 3. Discussion

The primary assumption of this article was, based on the most significant possible number of subjects, to extract new data on prehospital abnormalities in physical examination, which may indicate the need for urgent neurosurgical intervention. TBI remains the leading cause of death, long-term disabilities in previously healthy adults. Head trauma is one of the main causes of death in trauma patients. In North America, TBI kills 7 patients every hour [2, 4]. Although exact number is hard to determine, it is assessed that TBI affects 27–69 million people and their families every year [6, 7]. Due to the poorly explored effective treatments for TBI (most guidelines are based on weak data), surviving patients leave the hospital requiring many years of intensive rehabilitation, especially when they are in a minimally conscious state or in a persistent vegetative state [7]. According to a study conducted in 16 European countries, the number of years of life lost due to head trauma in Europe in 2013 was 1.3 million overall (1.1 million in males and 271,000 in females) [8]. From the perspective of head trauma patients, it is extremely important to initiate appropriate procedures to prevent secondary brain injury as early as possible. Maintaining normoxia and normotension are crucial. Pathophysiological reactions leading to posttraumatic coagulopathy (SWINE), fever, and glucose level fluctuations are other processes that threaten the development of secondary brain injury and decrease the chances of successful treatment completion. A single drop of systolic blood pressure <100 mmHg doubles mortality, and if SBP drops below 70 mmHg, it increases the death risk 6-fold [9].

The authors focused on the most commonly reported patient's complaints and on abnormal physical examination findings–apart from evident external symptoms of head trauma and a low GCS score–that may raise suspicion of head trauma requiring urgent neurosurgical intervention. Our analysis revealed few interesting insights, a lot corresponds to the data from most European countries, where a shift in the age of head trauma patients from young patients to those >65 years old has been observed in recent years [10]. In contrast with a paper by Bossers et al. [7], the most common cause of injuries in our study was falls on the same level. The incidence of serious injuries in the elderly is increasing, and the most common cause of injury is falls; falls are also the main cause of death in this group of patients [11]. Motor vehicle accidents were the most common cause of head trauma in a Dutch study but not even ranked among subjects who were qualified for urgent neurosurgical intervention in our study. All kinds of vehicles accidents were preceded by falls and resulted from epilepsy to beating. An aging society, the growing population of people who use various types of medications which affect blood clotting, polypharmacy, and the resulting falls all combine with poorly explored treatments for TBI to create a situation in which nearly one in five patients does not survive until discharge.

Currently, we do not have any effective prehospital tool for assessing patients' eligibility for urgent neurosurgical intervention. The GCS, as a simple and well-known scale, can yield widely varying scores and is useless after the administration of sedatives and analgesics–which is invariably part of the treatment in posttrauma patients–as well as in patients under the influence of alcohol. The pupillary assessment remains important, though 20% of the population has physiologic anisocoria and the clinical response to light may be disturbed by the administration of analgesics and sedatives. Also, the majority of people with posttraumatic anisocoria show no changes in CT. Norwegian HEMS project to implement CT scanner into their aircrafts, assays of SB100 in the patient's saliva or of the blood markers ubiquitin C-terminal hydrolase-L1 (UCHL-1) and glial fibrillary acidic protein (GFAP), or IT programs for evaluating indications for neurosurgical intervention, are unlikely to be introduced into the daily practice of prehospital teams for years to come [12–14]. Nonetheless, studies suggest that even basic prehospital emergency procedures in head trauma patients, such as ensuring appropriate oxygenation or maintaining normal pressure, are not performed correctly despite explicit guidelines [15]. In many countries, far-reaching attempts to prevent secondary brain injury are undertaken, such as smooth driving on the way to the hospital due to the likely negative impact of braking on intracranial pressure [16].

### 3.1. Future Research and Limitations

Our study on “Pre-hospital predictors for urgent neurosurgical intervention in the head trauma patient” yielded several intriguing findings. Confirmation of these findings through prospective studies conducted by other centres could potentially lead to the implementation of early response systems during the anamnesis collection stage. With the aid of computer systems, we can access information on the mechanism of injury, external injuries, and the patients' age, even at the dispatcher stage, which allows the neurosurgical facilities to be available when the patient arrives in the hospital. We do not believe the addition or introduction of a new scale or tool would benefit these patients. Our model demonstrated the feasibility of establishing an out-of-hospital early warning system.

The first limitation is that this is a retrospective study. The environment in the emergency department is one typically characterized by urgency, and so, medical personnel may not be able to identify all signs and symptoms of trauma during the data collection stage, especially during intensive and life-threatening situations, where time constraints would pose a threat to the patient's life. Although it is a retrospective study, we thoroughly reviewed each patient's record to ensure that no signs of trauma were missed. All data collected including ambulance cards, ED cards, and all neurosurgical records were carefully checked not to miss any signs of trauma, that could impact the results of our study, but of course, it can have some biases. Another limitation of this study was that it did not account for potential confounding variables that may have influenced the outcome, such as the severity of the trauma, comorbidities, or medications taken by the patient. Definitely, the lack of data on the medication taken is an important limitation of the study, and the intake of anticoagulants can certainly affect the prognosis. Undoubtedly, a significant limitation of the study stems from the inadequate access to data concerning the use of medications (specifically anticoagulants), chronic illnesses, surgical procedures, and the presence of cancer in the participants' medical histories.. However, from our experience in prehospital medicine, we often do not know the list of medications taken by unconscious patients. Are we able to determine the severity of the injury (how many stairs the patient fell down) of an unconscious patient found by his family at home? We are almost certain that at the prehospital and early-hospital level, it is necessary to simplify similar examinations as much as possible and the mechanism of injury and visible injuries must be enough for us to assess the patient because the rest can only be guessed at. Furthermore, this study was limited by the fact that is based only on two hospitals, although the two largest in our region. One was a large trauma centre and the second an emergency department which serves 60,000 patients/year. Moreover, the treatment strategies used in this study reflect the convention and tendencies used in our centres.

## 4. Conclusion

TBI remains a condition that devastates the lives of patients and their families. Effective treatments for TBI are disproportionately poorly explored. This study indicates many statistically significant demographic, mechanism of trauma, and abnormal examination findings in head trauma patients, which may suggest that transfer to a facility with a neurosurgery department is required as early as the prehospital care stage. While patients admitted to neurosurgery departments constitute a small percentage of TBI patients, this group is characterized by high mortality and their prognosis for normal performance status upon discharge is poor.

## Figures and Tables

**Table 1:** General characteristics of all head trauma patients.

Variables	Parameters	Overall (*N* = 7930)
Sex	Female	39.5% (*N* = 3134)
	Male	60.5% (*N* = 4795)

Age (years)	*N*	7903
	Mean (SD)	49.392 (20.861)
	Median (IQR)	47 (31–65)
	Range	5–104

Mechanism of trauma	Fall	46.4% (*N* = 3588)
	Road traffic collision	16.9% (*N* = 1304)
	Beating	13.2% (*N* = 1023)
	Hitting	9.4% (*N* = 725)
	Fainting	5.7% (*N* = 442)
	Epilepsy	5.7% (*N* = 438)
	Pedestrian hit by a car	1.9% (*N* = 143)
	Crushing between two objects	0.1% (*N* = 9)
	Kick	0.1% (*N* = 6)
	Head trauma during jump into the water	0.1% (*N* = 6)
	Bruised by an animal	0.1% (*N* = 6)
	Rape	0% (*N* = 1)
	Other	0.4% (*N* = 37)

Arrival on the day of injury	Yes	79.4% (*N* = 6300)
	No	20.6% (*N* = 1630)

Time from injury to arrival (days)	*N*	1625
	Mean (SD)	4.676 (12.102)
	Median (IQR)	2 (1–4)

Glasgow Coma Scale	*N*	5671
	Mean (SD)	14.799 (1.142)
	Median (IQR)	15 (15–15)
	Range	3–15

Loss of consciousness	Yes	42.2% (*N* = 2413)
	Recalls partially	0.5% (*N* = 26)
	No	57.3% (*N* = 3277)

Alcohol	Yes	21.4% (*N* = 1700)
	No	78.6% (*N* = 6230)

Alcohol (mg/dL)	*N*	440
	Mean (SD)	304.762 (107.588)
	Median (IQR)	306.5 (247–377.75)
	Range	20–592

Neurological abnormalities	Yes	7.1% (*N* = 551)
	No	92.9% (*N* = 7260)

Abnormalities found in physical examination	Yes	65.2% (*N* = 4955)
	No	34.8% (*N* = 2650)

Complaints	Yes	48% (*N* = 3649)
	No	52% (*N* = 3951)

The number of abnormalities found in physical examination	*N*	4955
	Mean (SD)	1.49 (0.855)
	Median (IQR)	1 (1-2)
	Range	1–10

The number of complaints	*N*	3649
	Mean (SD)	1.956 (1.045)
	Median (IQR)	2 (1–3)
	Range	1–10

Radiological examination (more than head CT scan)	Yes	59.4% (*N* = 4710)
	No	40.6% (*N* = 3215)

CT angiography	Yes	0.2% (*N* = 15)
	No	99.8% (*N* = 7915)

X-ray	Yes	44% (*N* = 3492)
	No	56% (*N* = 4438)

CT other than head CT	Yes	28.8% (*N* = 2282)
	No	71.2% (*N* = 5648)

Ultrasound	Yes	13.6% (*N* = 1075)
	No	86.4% (*N* = 6855)

Trauma CT	Yes	1.3% (*N* = 101)
	No	98.7% (*N* = 7829)

Classification	C1—urgent surgery	0.8% (*N* = 64)
	C2—delayed surgery	2.2% (*N* = 177)
	C3—24-hour follow-up	1.4% (*N* = 108)
	C4—no neurosurgical indication for hospitalization but patients were on anticoagulants, dangerous mechanism of trauma, were severely vomiting, confused, or/and without good family care	0.1% (*N* = 6)
	Abnormalities in radiological examinations other than head CT	18.8% (*N* = 1487)
	No abnormalities	76.7% (*N* = 6075)

Abnormalities in radiological examinations	Yes	23.2% (*N* = 1841)
	No	76.8% (*N* = 6089)

The number of abnormalities found in radiological examination	*N*	1841
	Mean (SD)	1.734 (1.381)
	Median (IQR)	1 (1-2)
	Range	1–13

Referral to another ward	Yes	11.2% (*N* = 887)
	No	88.8% (*N* = 7034)

Final patient status	Discharge	88% (*N* = 6969)
	Referral to another ward	8.5% (*N* = 672)
	Patient left the ward	1.5% (*N* = 120)
	Death	0.8% (*N* = 66)
	Patient did not give consent for hospitalization	1.2% (*N* = 94)

**Table 2:** Comparison of patient characteristic variables in relation to the variable: classification

Variables	Parameters	C1 (*N* = 64)	Others (*N* = 7853)	Test	*P* value
Sex	Female	32.8% (*N* = 21)	39.6% (*N* = 3110)	Chi-square	0.3276
	Male	67.2% (*N* = 43)	60.4% (*N* = 4742)		

Age (years)	*N*	64	7826	Mann–Whitney *U*	**<0.001**
	Mean (SD)	63.31 (21.79)	49.27 (20.82)		
	Median (IQR)	66.5 (41.5–81)	47 (31–65)		
	Range	18–95	5–104		

Time from injury to arrival (days)	*N*	16	1605	Mann–Whitney *U*	**0.0138**
	Mean (SD)	5.31 (4.61)	4.67 (12.17)		
	Median (IQR)	3.5 (2–6.25)	2 (1–4)		
	Range	1–14	1–240		

Glasgow Coma scale	*N*	47	5617	Mann–Whitney *U*	**<0.001**
	Mean (SD)	10.68 (4.64)	14.83 (1)		
	Median (IQR)	13 (5.5–15)	15 (15–15)		
	Range	3–15	3–15		

Loss of consciousness	Yes	67.7% (*N* = 21)	42% (*N* = 2386)	Fisher	**0.0184**
	Recalls partially	0% (*N* = 0)	0.5% (*N* = 26)		
	No	32.3% (*N* = 10)	57.5% (*N* = 3265)		

The bold values indicate significance.

**Table 3:** Detailed summary of the mechanisms of trauma in relation to patient classification.

Mechanism of trauma	C1 (*N* = 64)	Others (*N* = 7853)	*P* value^*∗*^
Fall	64.1% (*N* = 41)	45.1% (*N* = 3540)	**0.004**
Road traffic collision	4.7% (*N* = 3)	16.5% (*N* = 1298)	**0.017**
Beating	4.7% (*N* = 3)	13% (*N* = 1020)	0.074
Hitting	3.1% (*N* = 2)	9.2% (*N* = 723)	0.144
Fainting	4.7% (*N* = 3)	5.6% (*N* = 437)	0.975
Epilepsy	9.4% (*N* = 6)	5.5% (*N* = 431)	0.280
Pedestrian hit by a car	3.1% (*N* = 2)	1.8% (*N* = 141)	0.746
Alcohol	—	0.1% (*N* = 10)	1.000
Crushing between two objects	—	0.1% (*N* = 9)	1.000
Kick	—	0.1% (*N* = 6)	1.000
Head trauma during jumping into the water	—	0.1% (*N* = 6)	1.000
Bruised by an animal	—	0.1% (*N* = 6)	1.000
Rape	—	0% (*N* = 1)	1.000
Other	1.6% (*N* = 1)	0.3% (*N* = 26)	0.544

^*∗*^ *p* value of significance test for differences in proportions. The bold values indicate significance.

**Table 4:** Detailed summary of neurological abnormalities in relation to patient classification.

Signs	C1 (*N* = 41)	Others (*N* = 508)	*P* value^*∗*^
Aphasia	7.3% (*N* = 3)	2.2% (*N* = 11)	0.134
Aggression	2.4% (*N* = 1)	0.6% (*N* = 3)	0.701
Anisocoria	19.5% (*N* = 8)	19.7% (*N* = 100)	1.000
Asymmetry of the nasolabial folds	2.4% (*N* = 1)	0.4% (*N* = 2)	0.543
Incomprehensible speech	—	2% (*N* = 10)	0.764
No verbal response	14.6% (*N* = 6)	2.8% (*N* = 14)	**<0.001**
No response to pain	2.4% (*N* = 1)	—	0.105
No response to light	12.2% (*N* = 5)	2.6% (*N* = 13)	**0.004**
Patient on sedatives	4.9% (*N* = 2)	1.4% (*N* = 7)	0.290
Delirium	4.9% (*N* = 2)	0.4% (*N* = 2)	**0.022**
Patient on drugs	—	4.3% (*N* = 22)	0.344
Paresis	17.1% (*N* = 7)	8.1% (*N* = 41)	0.094
Amnesia	—	7.3% (*N* = 37)	0.143
Positive Babinski sign	9.8% (*N* = 4)	3% (*N* = 15)	0.065
Axial compression	—	1.4% (*N* = 7)	0.974
Decreased muscle tone in the limbs	2.4% (*N* = 1)	—	0.105
Lowered corner of the mouth	4.9% (*N* = 2)	0.8% (*N* = 4)	0.100
Nystagmus	4.9% (*N* = 2)	3.9% (*N* = 20)	1.000
Reduced response to light	—	1.8% (*N* = 9)	0.826
Dementia	—	2% (*N* = 10)	0.764
Agitation	7.3% (*N* = 3)	2.4% (*N* = 12)	0.169
Reduced response	2.4% (*N* = 1)	2.4% (*N* = 12)	1.000
Paralysis of the lower limbs	2.4% (*N* = 1)	—	0.105
Spasms	2.4% (*N* = 1)	—	0.105
Positive Romberg test	—	6.3% (*N* = 32)	0.190
Sleepiness	9.8% (*N* = 4)	2.8% (*N* = 14)	**0.049**
Confusion	14.6% (*N* = 6)	13% (*N* = 66)	0.953
Psychomotor retardation	4.9% (*N* = 2)	2% (*N* = 10)	0.503
Neck stiffness	4.9% (*N* = 2)	0.4% (*N* = 2)	**0.022**

The bold values indicate significance.

**Table 5:** Detailed summary of signs and symptoms in relation to patient classification.

Types	Complaints	C1 (*N* = 46)	Others (*N* = 7032)	*P* value
	Deformity of the nose	2.2% (*N* = 1)	0.2% (*N* = 13)	0.173
	Deformity of the clavicle	—	0.1% (*N* = 9)	1.000
	Deformity of the lower leg	2.2% (*N* = 1)	0.1% (*N* = 5)	**0.019**
	Bleeding from the ear	2.2% (*N* = 1)	0.2% (*N* = 16)	0.239
	Bleeding from the mouth	4.3% (*N* = 2)	0% (*N* = 3)	**<0.001**
	Bleeding from the nose	6.5% (*N* = 3)	1.1% (*N* = 80)	**0.007**
	Hematoma in the parietal region	2.2% (*N* = 1)	0% (*N* = 1)	**<0.001**
	Periorbital ecchymosis	10.9% (*N* = 5)	4.9% (*N* = 348)	0.134
	Numerous abrasions of the head	2.2% (*N* = 1)	0.2% (*N* = 13)	0.173
	Swelling and restriction of arm mobility	2.2% (*N* = 1)	0.5% (*N* = 37)	0.608
	Swollen face	2.2% (*N* = 1)	0.7% (*N* = 47)	0.735
	Abrasion of the forehead	2.2% (*N* = 1)	3.4% (*N* = 241)	0.953
	Abrasion of the temple	2.2% (*N* = 1)	0.7% (*N* = 47)	0.735
	Wound of the forehead	2.2% (*N* = 1)	6.7% (*N* = 471)	0.353
	Wound of the superciliary arch	—	5% (*N* = 355)	0.221
	Wound of the nose	—	2% (*N* = 139)	0.667
	Wound in the parietal region	—	4.9% (*N* = 345)	0.231
	Wound in the parieto-occipital region	—	1.1% (*N* = 80)	0.978
	Wound of the occiput	17.4% (*N* = 8)	5.5% (*N* = 388)	**0.002**
	Wound of the eyelid	—	1.2% (*N* = 82)	0.964
	Wound of the forearm	2.2% (*N* = 1)	0.2% (*N* = 17)	0.261
	Wound of the temple	8.7% (*N* = 4)	1.6% (*N* = 109)	**0.001**
	Wound of the lip	2.2% (*N* = 1)	1.3% (*N* = 88)	1.000
	Contusion of the shoulder	2.2% (*N* = 1)	0.1% (*N* = 7)	**0.049**
	Contusion of the forehead	8.7% (*N* = 4)	4.8% (*N* = 335)	0.369
	Contusion of the knee	2.2% (*N* = 1)	0.6% (*N* = 39)	0.636
	Contusion of the nose	—	1.9% (*N* = 131)	0.700
	Contusion of the parietal region	—	1.8% (*N* = 124)	0.730
	Contusion of the parieto-occipital region	4.3% (*N* = 2)	1% (*N* = 70)	0.128
	Contusion of the temporal region	4.3% (*N* = 2)	1.7% (*N* = 120)	0.422
	Contusion of the occiput	8.7% (*N* = 4)	2.1% (*N* = 145)	**0.009**
	Contusion of the eyelids	—	1.4% (*N* = 96)	0.874
	Contusion of the face	2.2% (*N* = 1)	1.1% (*N* = 75)	0.993
	Tenderness of the cervical spine	—	1.4% (*N* = 98)	0.862
	Tenderness of the occiput	—	1.2% (*N* = 82)	0.964
	Multisite injury	2.2% (*N* = 1)	1.8% (*N* = 124)	1.000
	Palpable skull fracture	6.5% (*N* = 3)	0.1% (*N* = 6)	**<0.001**
	Pain in the shoulder	—	3.9% (*N* = 275)	0.324
	Pain in the hip	2.2% (*N* = 1)	1.2% (*N* = 83)	1.000
	Pain in the abdomen	—	2.1% (*N* = 148)	0.633
	Pain in the cervical spine/neck	—	12.2% (*N* = 855)	**0.022**
	Headache	19.6% (*N* = 9)	32.6% (*N* = 2291)	0.085
	Pain in the chest	2.2% (*N* = 1)	3% (*N* = 211)	1.000
	Pain in the knee	—	2.3% (*N* = 163)	0.581
	Pain in the lower limb	—	1.5% (*N* = 104)	0.829
	Pain in the lumbosacral region	—	3.2% (*N* = 224)	0.419
	Pain in the elbow	—	1.2% (*N* = 87)	0.930
	Pain in the temporal region	2.2% (*N* = 1)	0.1% (*N* = 9)	0.087
	Pain in the back	—	1.2% (*N* = 84)	0.950
	Pain in the occiput	2.2% (*N* = 1)	0.3% (*N* = 20)	0.323
	Pain in the hand	—	2.4% (*N* = 172)	0.553
	Pain in the thoracic spine	—	1.7% (*N* = 117)	0.763
	Pain in the ribs	2.2% (*N* = 1)	2.8% (*N* = 194)	1.000
	Nausea	2.2% (*N* = 1)	5.7% (*N* = 402)	0.475
	Fainting after injury	2.2% (*N* = 1)	0.2% (*N* = 12)	0.151
	Sleepiness	2.2% (*N* = 1)	0.3% (*N* = 21)	0.343
	Vomiting	8.7% (*N* = 4)	3.8% (*N* = 268)	0.183
	Visual disturbances	—	1.5% (*N* = 108)	0.808
	Dizziness/balance disorders	4.3% (*N* = 2)	7.6% (*N* = 537)	0.576

The bold values indicate significance.

## Data Availability

The data used to support the findings of the study are available from the author upon request.

## Abbreviations

CT: Computed tomography
EBM: Evidenced-based medicine
ED: Emergency department
EMS: Emergency medical services
GCS: Glasgow Coma Scale
NS: Neurosurgery
RTC: Road traffic collision
TBI: Traumatic brain injury.
